# How to Shape Healthy Habits Within Pandemic-Related Constraints?

**DOI:** 10.3389/fpsyg.2021.711143

**Published:** 2021-09-03

**Authors:** Kamila Anna Dobrenko

**Affiliations:** Institut of Psychology, The Maria Grzegorzewska University, Warsaw, Poland

**Keywords:** positive health behavior change, habit (automatism), psychological need frustration, COVID-19, pandemic constraints

## Introduction

Incorporating healthy habits during the COVID-19 pandemic will help us to better survive this unusual time and may even stay with us for longer. Furthermore, new socialization, self-care, and exercise habits will continue to promote our health after the pandemic is over. While for many people the current situation is not very favorable, it is certainly quite new; as such, the environment is conducive to habit change (Duhigg, 2012; Ingram et al., 2020). It is up to each individual to decide whether or not to build healthier habits; however, in the context of the constraints imposed by the pandemic, most of us have had to significantly change our daily activities anyway (Maltagliati et al., 2021). An increasing number of people are seeking psychological help, and they link their reduced well-being directly to the pandemic imposing different ways of functioning than before. This reduced well-being is not surprising in specific crisis situations, such as job loss or health issues related to COVID-19. However, most people are simply uncomfortable with the long-term constraints of the pandemic. They find it difficult to organize their lives in the new conditions, including the ongoing satisfaction of their basic needs. This article explores the main areas of concern regarding the current constraints and lists ways to meet our most important psychological needs. The deprivation of these needs in periods longer than a few weeks is associated with negative consequences, and these consequences can already be observed.

In the first one and a half years of the pandemic, many published studies tackled the subject of the functional and psychological conditions of various groups affected by COVID-19 precautions. The most commonly reported symptoms were decreased sleep quality (Franceschini et al., 2020), changes in appetite (Shailaja et al., 2020), increased intake of alcohol and sleeping pills (Ingram et al., 2020; Pinkham et al., 2020), exacerbation of anxiety and depression symptoms, increased stress levels, and decreased well-being (Anyan et al., 2020; Shailaja et al., 2020; Paredes et al., 2021; Saddik et al., 2021). Many of these studies were conducted in the first few weeks of the lockdown, when the resources for coping with stress had not yet been depleted (de Zepetnek et al., 2021; Fernandez-Abascal and Martín-Díaz, 2021; Saddik et al., 2021). However, a study conducted on a large group of students in June and July of 2020 showed that, after several months of pandemic, the anxiety and depression indicators of the participants were almost twice as high as the corresponding values for the general population (Villani et al., 2021). Interestingly, people who were constantly coping with psychological conditions did not report any noticeable changes in symptoms related to mood shifts or even psychotic episodes from the period preceding the pandemic (Pinkham et al., 2020).

Psychological resilience is an important factor responsible for the reduction of the aforementioned effects of the COVID-19 pandemic (Anyan et al., 2020; Huffman et al., 2021; Kocjan et al., 2021; Paredes et al., 2021). Activities promoting health, concentrating on the formation of healthy habits such as physical activity, regular sleep patterns, or positive interactions with other people, can facilitate an increase in psychological resilience (Matvienko-Sikar et al., 2020; Brog et al., 2021; Strahler et al., 2021). Thus, the focus of the article is on the formation of attitudes that promote healthy behaviors in circumstances that are less favorable than usual.

## Why Are These New Modes of Functioning Difficult for Many People?

When the first lockdowns started in Europe in the spring of 2020, rapid and temporary adaptation to the new reality prevailed. Scientific journal databases already contained texts describing the negative consequences of pandemic-related constraints (Clemente-Suárez et al., 2020; Filipas et al., 2020; Franceschini et al., 2020; Gismero-González et al., 2020; Ingram et al., 2020; Shailaja et al., 2020).

As a psychotherapist, researcher, and psychologist supporting personality development, I wondered what might be helpful and accessible to those affected by the current situation. For about a year, I have been collecting ways to offset the negative consequences of the protracted pandemic. In this text, I share what tends to be difficult for the people I work with and what methods I have found helpful in coping with the constraints imposed by COVID-19. Most of the ideas listed here are easily accessible and relatively simple to implement independently.

### Change as a Source of Stress

Let me start by recalling a simple principle of the psychology of stress: Every change is a source of stress, and the bigger the change, the higher the level of stress that accompanies it (Terelak, 2008; Clemente-Suárez et al., 2020). It should be added that for many, pandemic stress has already crossed the threshold into chronic stress. This stress, which has persisted long enough to put a significant strain on our coping resources, can lead to health problems (Terelak, 2008).

Knowing that there is more stress now than before the pandemic, additional ways of coping can be implemented, and methods that have worked well so far can be intensified. We should also remember to think positively, which already reduces our levels of stress (Fredrikson, 2011). Other useful practices for stress relief include simple and varied relaxation exercises (e.g., focusing on relaxing specific parts of the body), physical activity (e.g., regular walks), meditation (e.g., basic mindfulness), and other activities that can serve the relaxation state (e.g., reading or knitting).

### Contact With Other People

We are social animals, as Aronson (2001) writes in the title of his book on social psychology. As a species, we are evolutionarily adapted to live in groups (Gazzaniga, 2011). According to Maslow's (Maslow, 2017) pyramid of needs, a person's social needs are just below their physiological and safety needs in terms of importance. This means that a person who is well-fed and well-rested and whose direct safety is not threatened needs socialization with other people to maintain a basic psychological balance.

A recent study found that the social support experienced by pregnant women during the pandemic was significantly lower than the support experienced by pregnant women before the pandemic (Matvienko-Sikar et al., 2020). This is an important observation, as the emotional state of a mother during pregnancy influences the development of the child in its fetal and neonatal periods (Schore, 1994; Grzegołowska-Klarkowska, 2018).

It is therefore advisable to at least look for substitute social supports. Telephone conversations with friends and virtual meetings in small groups are available options. We can also spend quality time with our family members or occasionally hang out with friends on a one-on-one basis. We should make sure that the time we spend together is productive. Conversations with others can be a good way of providing support in the current situation, as long as we avoid catastrophic themes—scaring each other (Terelak, 2008). These conversations should focus on sharing how to cope with the current circumstances, positive thinking, and humor.

Paradoxically, among members of a shared household, spending time apart can be a challenge. People differ in their needs to be alone; however, most people require some time for this kind of intimacy. Household members should avoid sharing the same room for 24 h a day, as this generates additional tension, especially in small spaces. Sometimes the need for solitude can be satisfied simply by going for a walk alone.

### Need for Stimulation

Working from home can mean spending a lot of time in a much quieter environment than the office. However, your home environment can be much louder if you have children who by nature make a lot of noise. In either situation, it is worth considering how your current level of stimulation—sensory impressions coming to you from the outside—compares to the level you are accustomed to, and how it relates to your personal preferences (Terelak, 2008; Cyniak-Cieciura et al., 2016).

The optimal state of stimulation for productivity can be defined by growth motivation theory in the context of “flow” (Csikszentmihalyi, 2005). Flow is characterized by the performance of tasks in an effective way. The flow state is found between boredom (when the level of stimulation is too low) and overload (when the level of stimulation is too high) (Katahira et al., 2018; Yazidi et al., 2020). Csikszentmihalyi (2005) theory can be useful, as it contains practical skill building suggestions related to gaining and prolonging a flow state. Such skills are commonly used by athletes trying to perform at their best. Flow is also increasingly recognized as facilitator of mental work (Katahira et al., 2018).

### Need for Activity

Due to remote work and the closure of leisure and entertainment facilities, many people have reduced their (already normally limited) physical activity (Maltagliati et al., 2021). In one study, about 50% of the respondents reported reduced physical activity, and only about 17% indicated an increase (Ingram et al., 2020).

Research findings suggest that there is a positive association between physical activity and psychological well-being (Filipas et al., 2020; Gismero-González et al., 2020; Ingram et al., 2020). Currently, the simplest and most available form of exercise is daily walking. It is also possible to exercise at home, supported by instructional workouts that can be easily found online.

### Need for Rhythm

People are by nature more or less rhythmic in terms of their bedtimes, meal times, and other daily routines (Cyniak-Cieciura et al., 2016). Under normal circumstances, we have far more rigid time frames imposed on us from the outside. Normally, most of our lives run according to a fixed schedule; however, remote working is characterized by much greater flexibility. When working from home, it can be difficult to separate the professional and personal spheres of life. Nowadays, many people are working, dealing with day-to-day household chores, relaxing, and finding entertainment within the same environment. Therefore, when working remotely, it is advisable to control office hours and avoid extending them.

Small rituals can help separate your work life from your home life, especially if the two share the same space. For instance, when you finish work, it is advisable to rearrange your surroundings slightly. These few minutes spent organizing your workplace in the morning and tidying it up in the afternoon are habitual signals similar to going to or leaving the office under normal circumstances. The function of such signals can also be fulfilled by going for a walk after work or performing any other form of activity clearly different from your work activities.

## How to Shape Healthy Habits?

Most of the things we do on a routine basis become automatic and habitual (de Houwer, 2019). Our brain looks for “shortcuts” in action patterns wherever possible. This saves energy for activities that cannot be done according to a well-worn pattern (Gazzaniga, 2013). Habits enable us to function efficiently. What we should know is that the automation process that underlies habit formation requires a lot of effort, but only for a short time—once a habit becomes automatic, it works on its own, with minimal effort from the subject (Jarymowicz and Ohme, 2002; Khaneman, 2011). Once trained, a habit can serve us for years if we find the time for it in our daily schedule.

We can distinguish two basic paths of habit formation. The first pathway is to change an existing habit into a new one that includes a routine we are interested in. The second pathway is to form a new habit where there was no existing custom. A third pathway, extinguishing a bad habit, should also be mentioned; it should be stressed that it is easier to swap an old habit for a new one than it is to extinguish an old habit entirely. By swapping a habit for a different one, we simply replace the routine when our minds are in a “what instead” mode. Here, I will focus on the first two paths mentioned, both of which can lead to the formation of healthy habits.

First of all, we need to consider the habits we want in our lives and whether they will actually serve us over a long period. This reflection is all the more important when considering that it is much easier to form a new habit than to get rid of it later. Secondly, developing a new habit must involve the emotional, cognitive, and physical spheres of motivation. It takes a lot of motivation to form a habit, and if we want something wholeheartedly, it is easier to strive toward (Jarymowicz, 2009; Jarymowicz and Szuster, 2017).

Our brain's process of habit formation constitutes a three-step loop of the components, cue, routine, and reward. A cue should be simple, clear, and unambiguous. A routine could be a habitual action that works to our advantage, e.g., an hour of physical activity, doing specific work tasks, household chores, or even creative work. The reward is usually the satisfaction of an important need (e.g., contact with other people, stimulation or activity). If a given action stimulates the reward system in our brain, there is good incentive to repeat the action in the future (Gazzaniga, 2013). This is why it is prudent to support the initial phase of habit training with additional external rewards that we find pleasant. However, external rewards should sporadic and in moderation so that they don't become an intrinsic part of the newly formed habit—the habit must provide gratification by itself.

Replacing an old habit with a new one may require careful self-observation and self-analysis to determine which important need was satisfied by the old habit and how it can be taken care of in the context of the new routine. While we are altering the routine, we can leave the old cue (e.g., the specific time or circumstance when the need appears), so that the way we satisfy the need becomes healthier (e.g., by eating something healthy instead of junk food). The reward (gratification) can also remain unchanged. If everything goes according to plan, the cue will be an effective signal to the brain as to which behavioral patterns it should trigger, and after the routine is completed, we will get the reward we need. The cycle will recur regularly. With time, the satisfaction resulting from the effects of the new habit will start to add to its total gratification (Duhigg, 2012).

We should often return to the question of why we want this habit in our life. It will remind us that the hardship of the present moment is part of a bigger plan, and this thought will stimulate self-motivation. The company of others is also helpful; being involved in a group that praises a particular habit or whose members already have it or want to work on building it together can be an additional, important source of motivation for us. In addition, we can support ourselves with modern technologies. There are more and more useful apps that work according to the habit formation process. These apps can remind us of our daily routine, give us praise for completing it, and monitor our progress.

## Discussion

The new circumstances in which many people are currently functioning due to the COVID-19 pandemic encourage the formation of new habits. This novel situation can be an opportunity to make small changes to the way we live our daily lives. In this way, firstly, we can better cope with the restrictions, and secondly, we can take better care of our health in the long term. It is advisable to concentrate new habits around the needs that are currently inadequately met: contact with other people, stimulation, activity, and rhythm. Finally, it is important to add to, review, and intensify our daily methods of coping with stress so that we can have moments of relaxation every day.

Habit formation is a simple mechanism that is based on the process of automation. To use it for our purposes, we must find a simple, unambiguous cue that will trigger a habit. Next, we must develop a desired routine that can be habitually followed. After that, we should clearly define the reward. Once such a cycle becomes automatic, the habit can work to our long-term advantage with minimal effort.

## Author Contributions

The author confirms being the sole contributor of this work and has approved it for publication.

## Conflict of Interest

The author declares that the research was conducted in the absence of any commercial or financial relationships that could be construed as a potential conflict of interest.

## Publisher's Note

All claims expressed in this article are solely those of the authors and do not necessarily represent those of their affiliated organizations, or those of the publisher, the editors and the reviewers. Any product that may be evaluated in this article, or claim that may be made by its manufacturer, is not guaranteed or endorsed by the publisher.

## References

[B1] Anyan F. Hjemdal O. Ernstsen L. Havnen A. (2020). Change in physical activity during the Coronavirus Disease 2019 lockdown in Norway: the buffering effect of resilience on mental health. Front. Psychol. 11:598481. 10.3389/fpsyg.2020.598481 33384645 PMC7770128

[B2] Aronson E. (2001). Człowiek Istota Społeczna [The Social Animal]. Warszawa: PWN

[B3] Brog N. A. Hegy J. K. Berger T. Znoj H. (2021). An internet-based self-help intervention for people with psychological distress due to COVID-19: study protocol for a randomized controlled trial. Trials 22:171. 10.1186/s13063-021-05089-9 33648555 PMC7917378

[B4] Clemente-Suárez V. J. Dalamitros A. A. Beltran-Velasco A. I. Mielgo-Ayuso J. Tornero-Aguilera J. F. (2020). Social and psychophysiological consequences of the COVID-19 pandemic: an extensive literature review. Front. Psychol. 11:580225. 10.3389/fpsyg.2020.580225 33391099 PMC7772398

[B5] Csikszentmihalyi M. (2005). Przepływ. Psychologia optymalnego doświadczenia [Flow. The Psychology of Optimal Experience]. Taszów: Biblioteka Moderatora.

[B6] Cyniak-Cieciura M. Zawadzki B. Strelau J. (2016). Formalna charakterystyka Zachowania – Kwestionariusz Temperamentu: Wersja Zrewidowana. Podrecznik [Temperament Questionnaire. Tekstbook]. Warszawa: Pracownia Testów Psychologicznych Polskiego Towarzystwa Psychologicznego.

[B7] de Houwer J. (2019). On how definitions of habits can complicate habit research. Front. Psychol. 10:2642. 10.3389/fpsyg.2019.02642 31849762 PMC6895142

[B8] de Zepetnek J. O. T. Martin J. Cortes N. Caswell S. Boolani A. (2021). Influence of grit on lifestyle factors during the COVID-19 pandemic in a sample of adults in the United States. Pers. Individ.Dif. 175:110705. 10.1016/j.paid.2021.110705 33531728 PMC7843028

[B9] Duhigg C. (2012). Siła nawyku [The Power of Habit]. Warszawa: Dom Wydawniczy PWN.

[B10] Fernandez-Abascal E. G. Martín-Díaz M. D. (2021). Longitudinal study on affect, psychological well-being, depression, mental and physical health, prior to and during the COVID-19 pandemic in Spain. Pers. Individ. Dif. 172:110591. 10.1016/j.paid.2020.110591 33518870 PMC7831714

[B11] Filipas L. La Torre A. Luzi L. Codella R. (2020). Born to run out of COVID-19: what gives us wings. Front. Sport Active Living 2:105. 10.3389/fspor.2020.00105 33345094 PMC7739669

[B12] Franceschini C. Musetti A. Zenesini C. Palagini L. Scarpelli S. Quattropani M. C.. (2020). Poor sleep quality and its consequences on mental health during the COVID-19 lockdown in Italy. Front. Psychol. 11:574475. 10.3389/fpsyg.2020.574475 33304294 PMC7693628

[B13] Fredrikson B. L. (2011). Pozytywność. Naukowe podejście do emocji, które pomaga zmienić jakość zycia [Positivity]. Poznań: Zysk i s-ka.

[B14] Gazzaniga M. S. (2011). Istota człowieczeństwa. Co sprawia, ze jesteśmy wyjatkowi [Human. The Science Behind What Makes Us Unique]. Sopot: Smak Słowa.

[B15] Gazzaniga M. S. (2013). Kto tu rzadzi – ja czy mój mózg? Co sprawia, ze jesteśmy wyjatkowi [Who's in Cherge?]. Sopot: Smak Słowa.

[B16] Gismero-González E. Bermejo-Toro L. Cagigal V. Roldán A. Martínez-Beltrán M. J. Halty L. (2020). Emotional impact of COVID-19 lockdown among the Spanish population. Front. Psychol. 11:616978. 10.3389/fpsyg.2020.616978 33391136 PMC7773811

[B17] Grzegołowska-Klarkowska H. J. (2018). Madrość osobowości: wspieranie rozwoju osobowości i zapobieganie jej zaburzeniom w całym cyklu zycia [The Wisdom of the Personality. Life Course Facilitation of Personality Development and Prevention of Personality Disorders]. Warszawa: ME-KOMP.

[B18] Huffman E. M. Athanasiadis D. I. Anton N. E. Haskett L. A. Doster D. L. Stefanidis D.. (2021). How resilient is your team? Exploring healthcare providers' well-being during the COVID-19 pandemic. Am. J. Surg. 221, 277–284. 10.1016/j.amjsurg.2020.09.005 32994041 PMC7486626

[B19] Ingram J. Maciejewski G. Hand C. J. (2020). Changes in diet, sleep, and physical activity are associated with differences in negative mood during COVID-19 lockdown. Front. Psychol. 11:588604. 10.3389/fpsyg.2020.588604 32982903 PMC7492645

[B20] Jarymowicz M. (2009). ‘'Racje serca i racje rozumu – w poszukiwaniu sensu idei powszechnie znanej,” in Nowe idee w psychologii [New Ideas in Psychology], ed Kozielecki J. (Gdańsk: Gdańskie Wydawnictwo Psychologiczne), 183–215.

[B21] Jarymowicz M. Ohme R. (2002). ‘'Homo sapiens czy Homo Automaticus?,” in Natura Automatyzmów [The Nature of Automatisms], eds Jarymowicz M. Ohme R. (Warszawa: Wydawnictwo Instytutu PAN Szkoła Wyzsza Psychologii Społecznej), 87–91.

[B22] Jarymowicz M. Szuster A. (2017). Nonspecific Impact of reflective mind on implicit evaluative processes: effects of experimental manipulations and selected dispositional factors. Front. Psychol. 8:1572. 10.3389/fpsyg.2017.01572 28955281 PMC5601000

[B23] Katahira K. Yamazaki Y. Yamaoka C. Ozaki H. Nakagawa S. Nagata N. (2018). EEG Correlates of the flow state: a combination of increased frontal theta and moderate frontocentral alpha rhythm in the mental arithmetic task. Front. Psychol. 9:300. 10.3389/fpsyg.2018.00300 29593605 PMC5855042

[B24] Khaneman D. (2011). Pułapki myślenia. O myśleniu szybkim i wolnym [Thinking, Fast and Slow]. Poznań: Media Rodzina.

[B25] Kocjan G. Z. Kavčič T. Avsec A. (2021). Resilience matters: explaining the association between personality and psychological functioning during the COVID-19 pandemic. Int. J. Clin. Health Psychol. 21:100198. 10.1016/j.ijchp.2020.08.002 33363581 PMC7753029

[B26] Maltagliati S. Rebar A. Fessler L. Forestier C. Sarrazin P. Chalabaev A.. (2021). Evolution of physical activity habits after a context change: the case of COVID-19 lockdown. Br. J. Health Psychol. 21, 1–20. 10.1111/bjhp.12524 33822454 PMC8250330

[B27] Maslow A. (2017). Motywacja i osobowość [Motivation and Personality]. Warszawa: Wydawnictwo Naukowe PWN.

[B28] Matvienko-Sikar K. Pope J. Cremin A. Carr H. Leitao S. Olander E. K.. (2020). Differences in levels of stress, social support, health behaviours, and stress-reduction strategies for women pregnant before and during the COVID-19 pandemic, and based on phases of pandemic restrictions, in Ireland. Women Birth. 1207, 1–8. 10.1016/j.wombi.2020.10.010 33162362 PMC7584422

[B29] Paredes M. R. Apaolaza V. Fernandez-Robin C. Hartmann P. Yañez-Martinez D. (2021). The impact of the COVID-19 pandemic on subjective mental well-being: The interplay of perceived threat, future anxiety and resilience. Pers. Individ. Dif. 170:110455. 10.1016/j.paid.2020.110455 33071413 PMC7552984

[B30] Pinkham A. E. Ackerman R. A. Depp C. A. Harvey P. D. Moore R. C. (2020). A longitudinal investigation of the effects of the COVID-19 pandemic on the mental health of individuals with pre-existing severe mental illnesses. Psychiatry Res. 294:113493. 10.1016/j.psychres.2020.113493 33038789 PMC7528831

[B31] Saddik B. Hussein A. Albanna A. Elbarazi I. Al-Shujairi A. Temsah M. H.. (2021). The psychological impact of the COVID-19 pandemic on adults and children in the United Arab Emirates: a nationwide cross-sectional study. BMC Psychiatry 21:224. 10.1186/s12888-021-03213-2 33941119 PMC8090921

[B32] Schore A. N. (1994). Affect Regulation and the Origin of the Self: The Neurobiology of Emotional Development. New York, NY: Norton.

[B33] Shailaja B. Singh H. Chaudhury S. Thyloth M. (2020). COVID-19 pandemic and its aftermath: Knowledge, attitude, behavior, and mental health-care needs of medical undergraduates. Ind. Psychiatry J. 29, 51–60. 10.4103/ipj.ipj_117_20 33776276 PMC7989454

[B34] Strahler J. Nater U. M. Skoluda N. (2021). Associations between health behaviors and factors on markers of healthy psychological and physiological functioning: a daily diary study. Ann. Behav. Med. 54, 22–35. 10.1093/abm/kaz018 31131391

[B35] Terelak J. F. (2008). Człowiek i stres [Man and Stress]. Bydgoszcz: Oficyna Wydawnicza Branta.

[B36] Villani L. Pastorino R. Molinari E. Anelli F. Ricciardi W. Graffigna G.. (2021). Impact of the COVID-19 pandemic on psychological well-being of students in an Italian university: a web-based cross-sectional survey. Global. Health 39, 1–14. 10.1186/s12992-021-00680-w 33823897 PMC8022300

[B37] Yazidi A. Mofrad A. A. Goodwin M. Hammer H. L. Arntzen E. (2020). Balanced difficulty task finder: an adaptive recommendation method for learning tasks based on the concept of state of flow. Cogn. Neurodynamics 14, 675–687. 10.1007/s11571-020-09624-3 33014180 PMC7501397

